# Evaluating community digital data linkage with or without community data use to increase antenatal care uptake in Western Kenya: protocol for a pragmatic open-label, cluster-randomised controlled superiority trial

**DOI:** 10.3389/frhs.2025.1697161

**Published:** 2026-01-27

**Authors:** Gerald Ong’ayo, Hellen C. Barsosio, Lilian Otiso, Alice Kamau, James Dodd, Linet Okoth, Mandela Oguche, Vicki Doyle, Eleanor Ochodo, Gordon Okomo, Feiko ter Kuile, Miriam Taegtmeyer

**Affiliations:** 1 Centre for Global Health Research, Kenya Medical Research Institute (KEMRI), Kisumu, Kenya; 2 Department of Clinical Sciences, Liverpool School of Tropical Medicine, Liverpool, United Kingdom; 3 LVCT Health, Nairobi, Kenya; 4 Capacity Development International, Liverpool, United Kingdom; 5 Department of Global Health, Faculty of Medicine & Health Sciences, Centre for Evidence-Based Health Care, Stellenbosch University, Cape Town, South Africa; 6 Department of Health, Homabay County Government, Homabay, Kenya

**Keywords:** community referral, data linkage, digital health, electronic community health information systems, electronic medical records, quality improvement

## Abstract

**Background:**

Less than 10% of pregnant women in Sub Saharan Africa achieve the World Health Organization recommended eight antenatal care (ANC) contacts for optimal pregnancy management. Robust strategies that involve community outreach programmes, integrated service delivery and continuity of care could help improve ANC uptake and quality. Kenya, as other countries, has promoted use of digital health records at the community and facility levels to improve quality and access to data and promote continuity of care. These records, however, are not always linked and access to data does not guarantee its use to drive quality improvement. C-it-DU-it (pronounced “See it, Do it”) is a two-arm pragmatic cluster-randomised trial set in Homabay County, Kenya. The trial will implement digital linkage of community and facility electronic patient data (control arm) and assess the impact of having quality improvement teams reviewing and acting on the linked data (intervention arm). While several areas are captured in the community health records, we will focus on uptake of ANC services as a lens.

**Methods:**

Eighteen healthcare facilities (clusters) will be randomly allocated to either the control or intervention arms at a ratio of 1:1. A data linkage module will be deployed in all clusters, enabling digital referral of pregnant women between the community and health facilities. In each intervention cluster, work improvement teams will be established and trained on reviewing these electronic ANC data, identifying problems, developing and deploying context-specific solutions to these problems and evaluating the impact of their interventions. ANC data will be extracted for 1,440 recruited pregnant women. The primary outcome will be the proportion of pregnant women with at least eight ANC contacts. Secondary outcomes will be ANC uptake before 16 weeks gestation, adverse pregnancy outcomes, uptake of required investigations, medication and skilled birth attendance.

**Discussion:**

This trial intends to generate evidence on the benefit of community work improvement teams to review and act on linked digital data to develop and deploy solutions to local problems. This strategy, if successful, will promote antenatal service uptake and quality resulting in improved pregnancy outcomes and progress towards sustainable development goals if appropriately scaled up.

**Clinical Trial Registration**: https://clinicaltrials.gov/study/NCT05929586, identifier NCT05929586.

## 1 Introduction

Sub Saharan Africa (SSA) carries the highest burden of maternal mortality, yet current estimates indicate less than 10% of the pregnant women meet the World Health Organization (WHO) target of eight antenatal care (ANC) contacts (1, 2). The Kenya Ministry of Health (MoH) adopted the WHO recommendation for eight or more ANC contacts in 2022 at a time when only 66% of the pregnant women in Kenya attained four or more ANC visits (3, 4). ANC attendance in low- and middle-income countries (LMICs), including SSA, is associated with reduced risk of neonatal mortality (5). Robust strategies are needed to improve ANC uptake if these and other benefits of increased uptake of skilled birth delivery and early postnatal care are to be achieved (6).

The WHO guidelines allow for context-specific variations on how the eight ANC contacts are achieved, including through community outreach programmes (3). Community-centred approaches such as community quality improvement (QI) have shown promise in strengthening community health systems in LMICs, particularly for maternal and child health outcomes (7). These QI interventions use a continuous QI model based on the “plan–do–study–act” cycle in which teams from the local community develop context-specific interventions which are tested and implemented after being proved to drive positive change (8, 9). This process is iterated to produce further improvements and the different QI teams share their learnings to promote best practice (9, 10).

A key component of QI approaches is access to reliable data to help identify gaps, set priorities and monitor progress in real-time (11). Digitisation of health data through introduction of electronic medical record (EMR) systems in healthcare facilities and community health information systems can provide more reliable and standardised data sources to support community QI initiatives (11). The Kenya MoH has been scaling up an electronic Community Health Information System (eCHIS) for Community Health Promoters (CHPs) in 2024 (12). This is a community health digital data capture tool designed to support household registration and service provision with in-built decision-support steps to facilitate referral to healthcare facilities and electronic reminders for community patient follow-up (12). In parallel, facility-based EMR systems, such as “KenyaEMR,” are being more widely used. The majority of these were initially used for vertical programmes such as HIV care delivery but may also support expanded use (antenatal care, delivery and postnatal care) (13). Community QI approaches that leverage these digital data systems could provide a robust health-systems-strengthening approach to improve ANC uptake and quality of care in Kenya and other LMICs.

The main barriers to attaining eight ANC contacts include financial constraints, rural residence, some religious affiliations and stigma towards unintended pregnancies (especially among adolescents). Others include cultural beliefs (such as pregnancies do n't need hospital attention), lack of partner support, distance to health facilities, high parity, lower level of education and delayed initiation of ANC (14–16). Context-specific interventions supported by accurate data, such as community QI initiatives leveraging digital health records, would afford robust strategies to improve ANC uptake in these circumstances. Evidence on these, however, is lacking as community health data digitisation at scale in SSA is a recent development.

C-it-DU-it (pronounced “see it, do it”) is a cluster-randomised controlled trial that will investigate the impact of having QI teams (subsequently referred to as “Work Improvement Teams” abbreviated as WITs) reviewing and acting on linked community and facility electronic data to improve ANC uptake.

## 2 Methods and analysis

### 2.1 Study aim

The C-it-DU-it trial aims to determine the impact on ANC uptake, ANC services and pregnancy outcomes of implementing WITs that use digitally linked data to identify and address local problems.

### 2.2 Study sites

The trial will be conducted in Homabay County in western Kenya, on the southern shore of Lake Victoria, approximately 370 km from the capital Nairobi. It has a population of 1.13 million, of whom approximately 24% are women of reproductive age (17). Close to 20% of pregnant women in Homabay are aged below 19 years and common preventable conditions including malaria, HIV and anaemia are major contributors to adverse birth outcomes (18). The County was the first to scale-up EMR systems for HIV care in its healthcare facilities (KenyaEMR) and will be one of the counties in which eCHIS adoption is accelerated.

The trial will be conducted in randomly selected healthcare facilities across the County based on the following criteria:

Government or faith-based facilities with ANC clinics
Facilities located in Homabay County mainland
Facilities having at least 80 attended births per year
ANC services linked with at least one fully functional community unit.

Referral hospitals, facilities located in Homabay County islands of Lake Victoria, facilities with very limited access and/or seasonal impassability, facilities declining to participate (as communicated by the facility in-charge) and facilities currently involved in another study targeting pregnant women directly (patient-level interventions) or indirectly (health system interventions) will be excluded.

### 2.3 Study design

This will be a two-arm, pragmatic, open-label, cluster-randomised superiority trial with 1:1 allocation of the arms.

### 2.4 Control (C-it) arm

All CHPs and most ANC facility staff currently use paper-based registers and referral forms. Plans by the MoH to deploy linked electronic data capture systems nationally, however, means digital data capture will be the standard of care in the lifetime of the trial. The control arm will therefore have digital data capture as the “enhanced standard of care” as follows:

Electronic data capture by CHPs via eCHIS during household visits.
Electronic data capture by ANC staff through use of the KenyaEMR Mother Child Health (MCH) module.
A linkage module linking KenyaEMR and eCHIS.

The county and sub-county health management teams will be supported by the trial team to distribute smart phones and ensure they are connected to the internet. They will work with the trial team to train CHPs in trial community units on the use of eCHIS for household registration, generation of National Unique Patient Identifier (NUPI), pregnancy mapping, patient referral and conducting household visits. NUPI refers to an identification number that will be generated from an individual's national ID or birth certificate number to enable linkage of an individual's clinical records across EMR systems. The same team will train ANC staff in trial sites on KenyaEMR MCH module to capture patient data, generate NUPI, identify community referrals and refer patients for community follow-up.

We will engage a software developer to design software in consultation with national and county health administration to link KenyaEMR and eCHIS records through the NUPI. This will enable longitudinal patient follow-up and prompts on eCHIS and KenyaEMR whenever a patient is referred. The software developer will offer technical support and address linkage errors such as NUPI mismatches in consultation with national and county health administration teams. We will train both CHPs and ANC staff at the facility on how to use the linkage module to anticipate and confirm completion of referrals. In order to comply with the Kenya Data Protection Act, we will implement additional access control measures including user authentication, role-based access and audit logs.

The use of eCHIS, KenyaEMR and the linkage app may be affected by system downtimes, electricity black-outs, disruption of internet access and loss or damage to equipment. To mitigate these, trial staff, ANC staff and CHPs will be trained to safely handle the equipment, troubleshoot and contact the right people when facing technical challenges. Since eCHIS roll-out will be country-wide, we anticipate provision for internet access for the CHPs to allow (near) real-time data uploads. Where not available, the trial team will provide back-up internet access within trial sites.

Finally, we will train both CHPs and facility staff on the trial definitions of community ANC contacts, the services offered to women in the immediate post-partum period and how to fill the trial CHP case report form (CRF) to document home visits. CHP training will include instructions on how to provide ANC-related services such as screening for danger signs, measuring blood pressure, mid-upper arm circumference and temperature, conducting pregnancy and malaria rapid diagnostic tests, and checking adherence to mebendazole, Iron and Folate Supplements and Intermittent Preventive Treatment for malaria in Pregnancy (IPTp). The CHP CRF will be attached to the MCH Book carried by the pregnant women.

### 2.5 Intervention (C-it-DU-it) arm

Intervention sites will implement WITs in addition to adopting the enhanced standard of care (C-it). Each community unit will have one WIT comprising 3–4 CHPs, a Community Health Assistant (CHA), an ANC staff and/or the healthcare facility in-charge from the link facility (facility to which the CHPs refer patients from the community), a data clerk, an adolescent mother and a community representative. We will use a continuous QI model involving iterative cycles of developing and testing interventions with periodic collaborative sessions for sharing their innovations.

**Figure 1:**
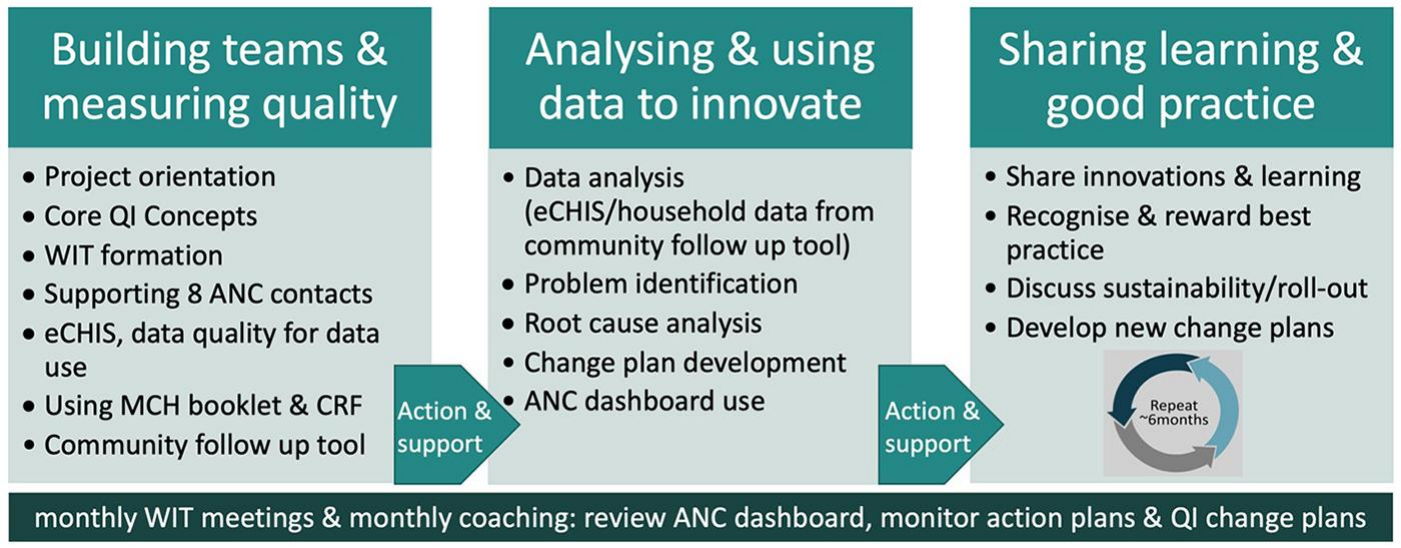
Summary of the phased WIT training model.

A five-person facilitation team [from Capacity Development International (CDI), LVCT Health and representatives from the sub-county and County Health Management Teams] will train the WITs in groups small enough to check learning and ensure fidelity to content. We will use a phased training approach, described in Figure 1, building on previous experience embedding QI into community health (9).

#### 2.5.1 Building teams and measuring quality

The facilitation team and respective sub-county, facility and community unit leads will agree on WIT membership. They will orient WIT members on their roles, core QI concepts and agree on their terms of reference. They will discuss the changes in government policy from four to eight ANC contacts and the role of CHPs in early pregnancy identification and supporting pregnant women to achieve eight ANC contacts. They will train WITs on use of a community follow-up tool (9) to obtain insights into the quality of CHP community ANC visits.

There will be a 10-week interval during which WITs will sensitise link facility staff, CHPs and community members about their role in improving ANC uptake. CHAs will encourage regular household visits and assess eCHIS and CHP CRF data during monthly supervision meetings. The facilitation team will support the WITs to identify and train selected community volunteers to visit a sample of households to administer the community follow-up tool (9).

#### 2.5.2 Analysing and using data to innovate

WITs will convene to share learning and experiences in implementing their action plans from the first training. The facilitation team will train WITs on use of tables, graphs and dashboards for data presentation and tracking of indicators while adhering to the Kenyan Data Protection Act 2019 (19). WITs will analyse eCHIS data, facility data and community follow-up tool data to identify and prioritise quality problems and develop a clearly defined problem statement. They will conduct a root cause analysis using the “Five-Whys?” technique (20) and brainstorm potential solutions with the aim of developing a change plan to address root causes. Sub-county health administration staff will be trained as WIT coaches.

There will be a 5–6-month interval during which WITs will implement and monitor their change plans. WITs will meet monthly to implement and refine their solutions, monitor core ANC indicators using their dashboards and prepare story boards to present to County officials during learning events. Coaches from the facilitation team and sub-county will offer mentorship to promote WIT team adherence to their mandate.

#### 2.5.3 Sharing learning and good practice

Learning events will take place every 6 months and will convene all WITs, the facilitation team and county health administration. This will allow showcasing and rewarding of best practices and provide a forum for advocacy with higher levels of the healthcare administration system.

#### 2.5.4 Definition of full implementation and embedding of the intervention

**Figure 2:**
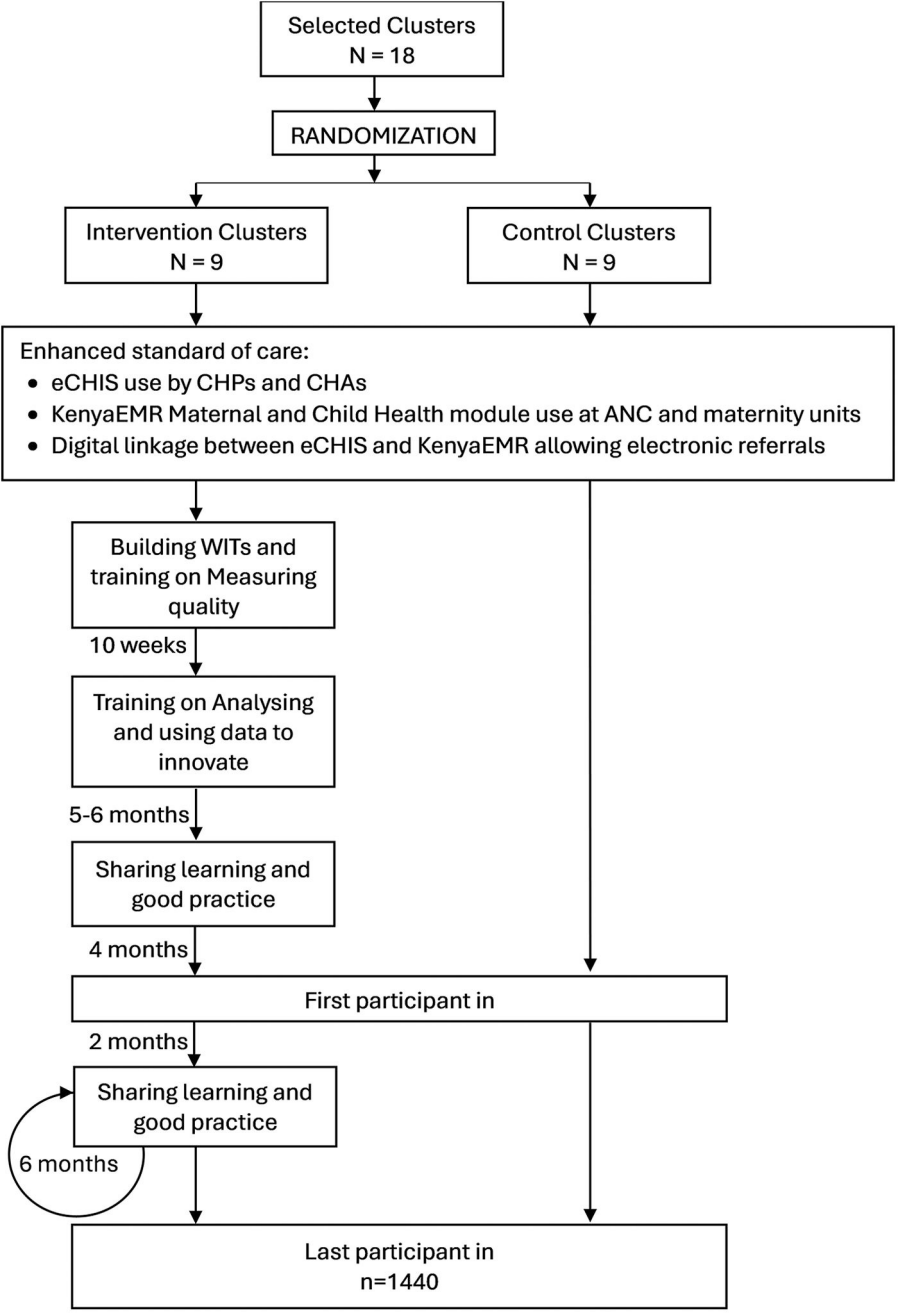
Summary flow diagram indicating project activities across arm and timelines. ANC, antenatal care; CHAs, community health assistants; CHPc, community health promoters; eCHIS, electronic community health information system; WIT, work improvement team, *N*, number of clusters; and *n*, number of participants.

Full implementation will be assumed to occur after the training on analysing and using data to innovate is completed. Full embedding of the intervention will be assumed to occur 3–6 months after this. The intervention's earliest ANC contact likely to be impacted will deliver approximately 7–9 months after embedding (assuming ANC contact occurs in the first trimester). Outcome assessment (first patient-in) will therefore start 10 months after full implementation (Figure 2).

### 2.6 Relevant concomitant support

Routine supervisory activities by sub-county and county health management team members will continue in all sites during the implementation of the trial. The county staff are central to the design and delivery of the intervention and we will work with CHPs, facility staff and county health management without offering additional remuneration for implementing the enhanced standard of care or community QI.

### 2.7 Cluster definition, randomisation and blinding

We will define a cluster as an ANC clinic meeting selection criterion, and this will be the unit of randomisation. An independent statistician will use a covariate-constrained randomisation approach to achieve balance across the two study arms. Specifically, we will apply the *l2* balance metric implemented in the “**cvrall**” package in R statistical software. All possible allocation schemes will be evaluated against predefined balance criteria, and a final allocation will be selected at random from a constrained set. A fixed random seed will be used to ensure reproducibility of the randomisation procedure. These covariates include sub-county, number of functional community units, number of CHPs, number of women of childbearing age, service provision level and the number of women attending four ANC visits and eight ANC visits.

We will convene the county and sub-county leadership, facility representatives and community representatives in a public event during which three County leaders will be asked to pick an unmarked sealed envelope containing details of allocation to a randomisation arm. We are unable to blind the allocation of arms given the nature of the intervention but will use a 5 km buffer zone between intervention and control facilities to minimise risk of contamination.

To minimise bias, we will use an objective primary outcome measure, and the statistician will be blinded regarding the allocation arm when the statistical analysis plan (SAP) is developed and analytical syntax is written. The actual allocation will only be provided to the statistician after locking the database and approval of the SAP by an independent Data Management Committee (DMC).

### 2.8 Sample size and study endpoints

We conducted sample size calculations with consideration for the primary endpoint using Power Analysis Sample Size Software (PASS) v20 (21). The primary objective is to determine if C-it-DU-it is superior to C-it in increasing uptake of eight ANC contacts in western Kenya.

#### 2.8.1 Primary endpoint

The primary endpoint will be the proportion of pregnant women attaining at least eight ANC contacts defined as either a scheduled ANC visit in the facility or a scheduled ANC contact with a CHP in the community assessed at birth (or within the first 6–8 weeks for home births). Community ANC contacts will be defined as a home visit that covers the elements of a community ANC visit plus at least one diagnostic test or the distribution of at least one drug. Diagnostic tests or drugs administered include pregnancy tests, malaria tests, iron and folate supplements, sulphadoxine-pyrimethamine, co-trimoxazole and mebendazole.

**Figure 3:**
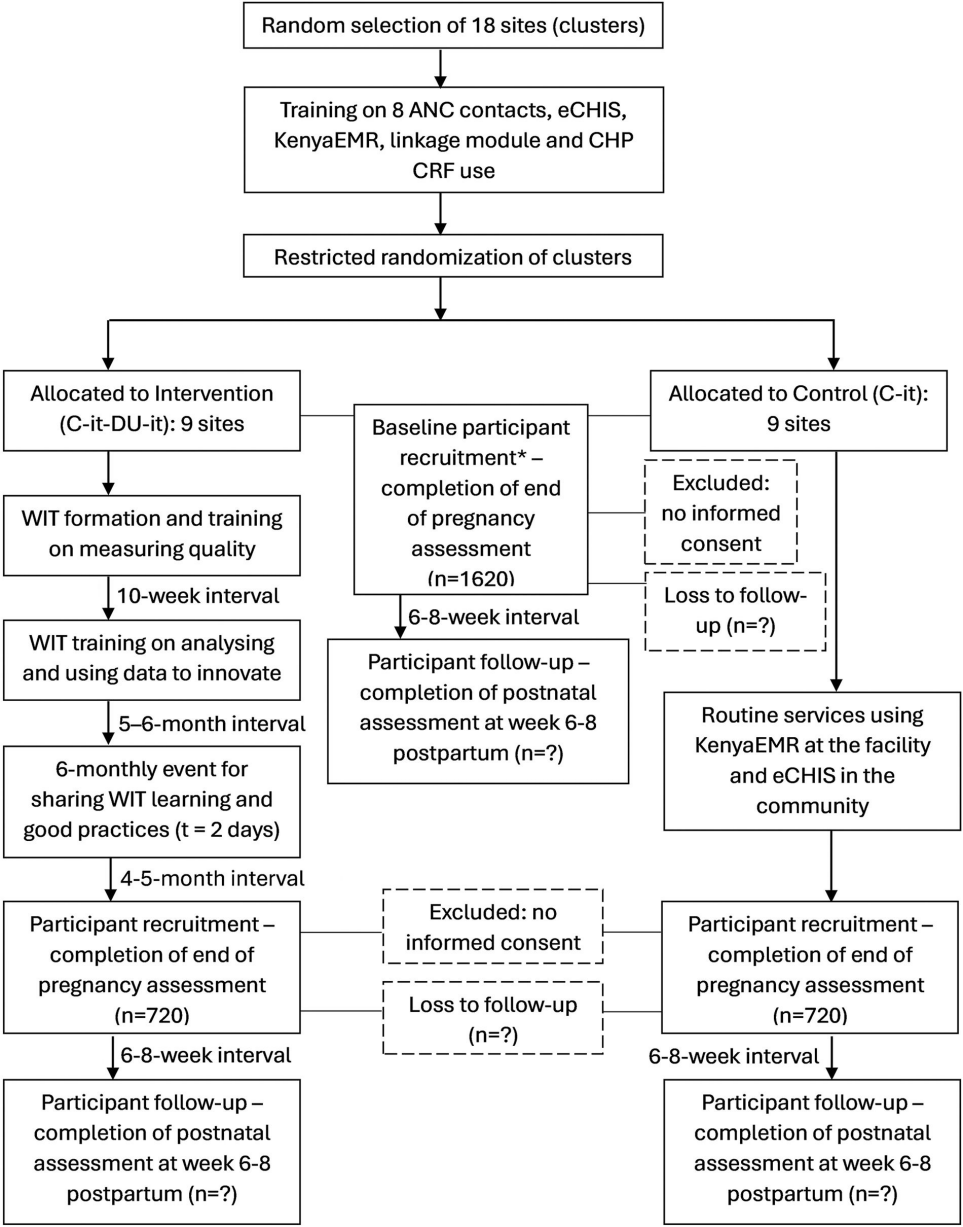
Implementation and participant timeline. The asterisk denotes baseline participant recruitment and follow-up will continue as the intervention is being implemented. eCHIS, electronic Community Health Information System.

During a baseline survey, we estimated 3% of the pregnant women in Homabay achieved eight ANC contacts and deemed an increase to 12% would be the minimum effect size to warrant national scale-up of the intervention. To detect this four-fold increase in the primary outcome assuming an intra-cluster correlation coefficient (ICC) of 0.025, 90% power and an alpha of 0.05, a sample size of 1,024 women in 16 clusters (8 per arm) would be required giving an average cluster size of 64 women. To account for efficiency loss due to cluster size imbalance, we increased clusters to 9 per arm resulting in a total of 1,152 participants. To adjust for anticipated 20% loss to follow-up, we increased the sample size estimate to 1,440 participants in total (720 per arm) giving an average cluster size of 80 women. The sample size calculation will be reviewed and updated (if necessary) after completion of baseline data collection from 1,620 participants in trial sites to obtain an updated ICC. Baseline data collection will take place in the months during which WIT training and embedding of the intervention will occur, prior to recruitment of the first patient-in (Figure 3).

#### 2.8.2 Secondary objectives and endpoints

The secondary objectives are:

- To determine if C-it-DU-it is superior to C-it on other secondary endpoints of ANC uptake. The endpoints are the proportion of women who had at least four scheduled ANC visits in the facility; the proportion of women who had at least eight scheduled ANC visits in the facility; the frequency of scheduled ANC visits in the facility; and the frequency of ANC visits in the community.
- To determine if C-it-DU-it is superior to C-it in increasing early ANC attendance with the endpoint of the proportion of women with a first ANC contact before 16 weeks gestation.
- To determine if C-it-DU-it is superior to C-it in improving the quality of ANC with the endpoints of the proportion of women who received at least three courses of IPTp-SP, iron and folate supplements for 90 days, testing and management for HIV, testing and management for malaria, testing and management for syphilis, and testing and management for anaemia.
- To determine if C-it-DU-it is superior to C-it in increasing uptake of skilled birth attendance with the endpoint being the proportion of women who had a skilled birth attendance.
- To determine if C-it-DU-it is superior to C-it in reducing the risk of adverse pregnancy outcomes with the endpoints being the proportion of women with a composite of foetal loss (stillbirth or spontaneous miscarriage), low birth weight or neonatal mortality.
- To determine if C-it-DU-it is superior to C-it in increasing uptake of week six childhood immunisation.

#### 2.8.3 Exploratory endpoints

In addition, we included the following two additional exploratory endpoints:

- To determine if C-it-DU-it is superior to C-it in improving uptake of all four ANC tests as measured by the proportion of women who received testing and management of HIV, syphilis, malaria and anaemia.
- To determine if C-it-DU-it is superior to C-it in improving uptake of HIV prevention services as measured by the proportion of women who received testing and management for HIV in the third trimester or at delivery, and the proportion of HIV-exposed infants with negative HIV PCR DNA tests at 6–8 weeks postpartum.

### 2.9 Inclusion and exclusion criteria for pregnant women

The trial population will consist of pregnant women, regardless of age, who are resident within the catchment areas of trial sites for the duration of their pregnancy (and the trial period) and have delivered recently (within the preceding 8 weeks), who are willing to participate and provide written informed consent. Women enrolled in other interventional studies targeting pregnant women will be excluded.

#### 2.9.1 Participant screening

Trial data clerks and fieldworkers will screen women for eligibility at delivery at the maternity wards, post-natal clinics, child welfare clinics, female adult wards, gynaecology wards and outpatient clinics to allow capture of adverse pregnancy outcomes and early pregnancy losses. Trial fieldworkers will conduct home visits to screen eligible women who delivered at home or in non-study sites within the first 8 weeks post pregnancies. Trial data clerks will contact participants when the follow-up window opens (6–8 weeks post pregnancy) to minimise loss to follow-up.

### 2.10 Sources of data

The primary data source will be the mother and child health book and the CHP case report form. Health facility registers will be the secondary source, and patient interviews, the tertiary source.

#### 2.10.1 The MCH book

All mother and child health services including routine visits during and after pregnancy, visits due to illness and visits for vaccination are documented in the MCH book (also known as MoH 216 or the ANC book). These books are printed by the government and issued to each pregnant woman free of charge during their first ANC visit. Women are expected to carry them for every subsequent visit until their child attains 5 years of age. Data extracted from the books will include last menstrual period, number of ANC visits, treatment of medical conditions, ANC profile test results, mode of delivery, delivery outcomes, newborn health at delivery, birth weight and prevention interventions given (including insecticide treated bed nets, IPTp-SP, iron and folate supplements and Tetanus Toxoid vaccinations).

#### 2.10.2 The community health promoter case report form

The trial designed a CHP CRF attached to the MCH books issued within the trial catchment area. It contains a section to be filled by CHPs during home visits to document the ANC and PNC-related services they offered and a section for ANC staff to comment and sign off on the information entered by CHPs. This will enable the mother to have a record of all services received during the antenatal and immediate postnatal period.

#### 2.10.3 Participant interviews

Trial data clerks and fieldworkers will interview women at delivery and the 6–8-week postpartum period. Data collected will cover demographic details, socio-economic status, treatment of common conditions during pregnancy, place of delivery (health facility or home), type of delivery (vaginal or caesarean section), complications during delivery, the health of the baby at delivery, lactation history, use of prevention interventions (such as bed nets, malaria chemoprophylaxis, and iron and folate supplements), care-seeking behaviour, past obstetric history and access to care.

#### 2.10.4 Healthcare facility registers

Trial data clerks and fieldworkers will abstract participant data from other clinic and ward registers to corroborate participant interviews and data missing from MCH books. These data include pregnancy outcomes, complications at delivery and other newborn morbidity outcomes. These registers include maternity, adult ward, gynaecology ward, out-patient department and child welfare clinic registers.

### 2.11 Data capture, storage and curation

Data clerks will enter de-identified participant data on REDCap using internet-enabled tablets for encrypted data transfer to a secure, shared drive managed by Kenya Medical Research Institute (KEMRI) and backed up on a secure server at LSTM, UK. They will maintain a separate participant link log with participant identifiers accessible to the data clerks and data manager for query resolution.

Data validation will be completed by a data manager who will work with the trial statistician to develop syntax-driven consistency checks and data cleaning steps. Once cleaned and locked, the trial statistician will create final variables for analysis. The final cleaned database will be available in SAS, STATA and SPSS formats. A REDCap data dictionary of the raw database will be available, and the data dictionary of endpoints and other derived variables will be provided on request.

### 2.12 Analytical plan/statistical methods

The intention-to-treat analysis population will consist of all eligible recruited participants who consented to participate in the trial from all randomised clusters. The per-protocol analysis populations will be those eligible, recruited and consented whose clusters had full implementation after WIT training on “analysing and using data to innovate” was completed within the expected timelines (Figure 2). These WITs will have determined a problem statement, developed a change plan, implemented it and tracked the relevant indicators in their monthly meetings.

No formal interim analysis will be carried out due to the long period between exposure to intervention and outcome ascertainment, and because this study does not test a direct patient-level intervention. The Institutional Review Boards (IRBs), the DMC and the Trial Steering Committee (TSC) will review study enrolment, progress and any safety concerns arising while the study is ongoing. Therefore, we will not define a stop criterion for this trial based on interim analyses. Instead, the trial will be stopped due to any major safety concerns, including direct harm to participants, as agreed upon by the IRBs, DMC and TSC.

We will conduct the primary analysis on the intention-to-treat population (ITT) using a binomial multilevel (random effects) model with a logit-link for estimation after which risk ratios (RR) and 95% confidence intervals will be estimated using parametric bootstrap procedures. This model was chosen as directly estimating RR is less robust, because RR may vary for reasons other than the magnitude of the effect because it is a ratio of two posterior probabilities, both of which are dependent on baseline prevalence of an outcome (22).

We will incorporate the facility (cluster) as a random effect and estimate *P*-values using parametric bootstrap procedures. We will assume the structure of the variance–covariance matrix for the random effect to be exchangeable. We will use similar methods of analysis to obtain crude and adjusted proportions for secondary outcomes and estimate standard errors of effect estimates while accounting for cluster-level correlation in all analyses.

Covariate analyses will be performed on the primary outcome on the ITT population, using baseline values: age (continuous, years), gravidae (paucigravidae (1st or 2nd), multigravidae (3rd+), education (primary not completed, primary, secondary, post-secondary) and marital status (single, married/co-habit, divorced/separated, widow). Pre-specified subgroup analyses will be performed for the primary outcome on the ITT population on stratification and baseline covariate variables, with age dichotomised into “lesser than” or “greater than” median. The treatment effect within each category of these variables will be estimated, and the interaction effect between treatment and each variable will be assessed to explore effect modification.

Missing baseline co-variables (as defined in the SAP prior to data lock) will be imputed using simple imputation methods based on the co-variable distributions, should the missing values for a particular co-variable be less than 5%. If the missing values for a covariable are ≥5%, then they will be imputed using Markov chain Monte Carlo (MCMC) methods. In the event of imputation for missing values used in primary or secondary outcome analyses, sensitivity analyses using complete cases will also be carried out.

We will not use imputation when missing outcome data arises due to incomplete data in MCH books and will report the fraction of missing outcome assessments by study arm for the intention-to-treat study population.

For secondary outcomes, confidence intervals and *P*-values will not be adjusted for multiple testing. However, care will be taken when interpreting the findings from these analyses. Subject to journal requirements, the following statement will be added to the figure or table notes comparing multiple secondary outcomes: “The *P*-values and widths of the confidence intervals have not been adjusted for multiplicity, so should not be used to infer definitive treatment effects for secondary outcomes.”

### 2.13 Public and stakeholder involvement

The trial was co-developed with the MoH (Division of Reproductive Health and Division of Community Health) and County government health administration. We collectively chose the primary outcome (eight ANC contacts) based on changes in national policy (4) and agreed on the definition of community ANC contacts and the design of the CHP CRF. We selected the electronic data capture tools (eCHIS and KenyaEMR) in line with MoH priorities for national scale-up. County and sub-county administration members will form part of the team training ANC staff and CHPs in trial sites and offering support supervision to WITs in intervention sites. County and sub-county leadership, facility representatives and community representatives will be present during allocation of clusters to intervention/control arm.

## 3 Ethics, oversight and dissemination

The trial will be conducted in compliance with the principles of the Declaration of Helsinki (1996), the principles of Good Clinical Practice and in accordance with regulatory requirements in Kenya. Trial staff will explain the trial's purpose to each potential participant and clearly communicate that participation is voluntary and will not affect their access to services at the facility before participants are asked for signed informed consent. Participants unable to read and write will be requested to provide oral consent and a thumbprint in the presence of an impartial witness. Women aged below 18 years who are pregnant or have children are considered “mature minors” in Kenya and will be able to consent per KEMRI-Scientific and Ethics Review Unit guidelines.

A TSC will provide overall oversight and will comprise of at least 75% independent members. They will advise on all aspects of the trial, monitor progress, adherence to protocols and ethics, and agree on any substantive protocol amendments. They will ensure patients' rights, safety and well-being are upheld and align with the safeguarding approach. A DMC consisting of four independent members (a chair, a topic expert, an obstetrician and a biostatistician) will periodically review and evaluate the accumulated study data for participant safety, study conduct, progress and efficacy and give recommendations to the investigators. The DMC will also review and approve the statistical analysis plan which will be implemented by the trial statistician. Trial results will be published in peer-reviewed journals with equitable authorship governed by International Committee of Medical Journal Editors criteria. Study staff will present results during community meetings in the trial sites and in conferences.

## 4 Discussion

The C-it-Du-it trial will provide evidence on the impact of community QI interventions using digitally linked community and facility data in a real-world health systems context. The push to digitise patient data at the facility and community levels has the potential to improve patient outcomes through improved data quality and data access, enabling the tracking of individual pregnant women. While donors and governments rush to digitise and link health data, we do not currently know if digital data linkage alone will work to achieve health outcomes or if linkage plus structured community data use is necessary.

Previous studies linking ANC data in LMICs have shown QI methodologies are rarely adapted for use beyond traditional healthcare facilities with limited implementation in resource-poor settings (23). In this study, we have worked with nationally adopted EMR systems at community and facility levels, added bidirectional eCHIS–EMR linkage, adapted QI training to community teams, developed community dashboards and included a focus on adolescents. We anticipate a causal pathway to increased ANC uptake. In our case, the data linkage should give more visibility of missed/late pregnancies leading to targeted CHP follow-up and increased ANC uptake. WITs can work together to identify problems from the data (such as late presentation among adolescents), investigate the root cause (e.g., school dropout, stigma, etc.), develop a targeted intervention (e.g., with schools, families and communities) and efficiently monitor progress on that metric.

Our trial implements a pragmatic design with integration into the existing health system. The trial was co-developed, and will be co-implemented, with national and County health administration to ensure alignment with government priorities and increase the likelihood of adoption and scale-up. We are aware county activities that convene ANC staff and community health staff could create opportunities for contamination through WIT members interacting with staff from control sites. Likewise, county staff offering support supervision and coaching to WITs may inadvertently influence operations at control sites within their jurisdiction. To minimise this, the concern will be raised, addressed and re-enforced by the County during the intervention team training sessions. Service delivery could be affected by stock outs of ANC medication, testing equipment and MCH books. These could discourage attendance to ANC clinics and directly impact the primary outcome. To mitigate this, the trial team will discuss with the County administration on the importance of maintaining stocks in the healthcare facilities and will provide a buffer stock of mebendazole, iron and folate supplements, IPTp-SP, pregnancy test kits, malaria rapid test kits and MCH books at all the trial sites.

Since we anticipate the trial may have unintended consequences such as increased documentation burden on health facility staff having to fill paper registers in addition to using the EMR system, we will advocate with county health management to allow EMR-generated electronic reports during the trial period. Increased ANC attendance in the intervention arm may result in increased detection of adverse pregnancy outcomes without a true increase in the incidence/risk. We will acknowledge this limitation in our interpretation of findings.

QI and health systems strengthening is often assessed by process evaluations and other operational research study designs leading to concerns from policymakers about the strength of evidence. We have risen to this challenge with a protocol suited to the health systems reality in Homabay. In addition to our pragmatic design and strong government ownership, our trial has several other strengths. We cite the Kenya Data Protection Act and echo here how governance, consent and NUPI linkage risks are mitigated through role-based access, audit logs and reconciliation procedures. We have an objective primary outcome, a pre-specified SAP and a blinded analyst.

We are aware that even once trial results are available, policymakers may still have concerns around the real-world implementation of our findings. For example, they may question contextual factors around the effectiveness of WITs and worry about implementation costs. We have therefore nested a realist evaluation, a health-economics evaluation (from a societal and health system perspective) and a realist economic evaluation into the study.

The trial was co-developed and will be co-implemented with national and county health administration to ensure alignment with government priorities, enable understanding and ownership of the intervention, and increase the likelihood of adoption and scale-up.

### 4.2 Trial status

Baseline data collection begun in December 2023, WIT formation and trainings were completed in April 2024 with the first learning event being held in October 2024. Trial participant recruitment for outcome assessment begun in February 2025 and will continue until February 2026, or until the target sample size is achieved (if earlier).

Protocol Details:

Version 2.0
Issue Date: 15 February 2023
Protocol Amendment Number: 01
Revision Chronology:
  ○ 22 December 2022: Original
  ○ 15 February 2023: Amendment 1

Primary reason for amendment—removal of the previous standard of care arm due to rapid adoption of electronic community health information system across the country resulting in an anticipated change of standard of care from paper to electronic records in the study sites.

Additional changes—clarify definition of primary outcome (ANC contact), addition of a case report form to help capture community ANC contacts, updated sample size calculation, updated site selection criteria, revision of secondary outcomes, addition of exploratory outcomes, addition of baseline data collection, revision of timeframe to allow roll-out of electronic community health information systems before implementation of the intervention and clarification of participant reimbursement process.

All future amendments will be submitted for approval by KEMRI Scientific and Ethics Review Unit and the Liverpool School of Tropical Medicine's Research Ethics Committee.
