# Supplementary material for: Evaluating community digital data linkage with or without community data use to increase antenatal care uptake in Western Kenya: protocol for a pragmatic open-label, cluster-randomised controlled superiority trial
Source: Front Health Serv. 2026 Jan 27;5:1697161. doi: 10.3389/frhs.2025.1697161 (PMC12886392; doi:10.3389/frhs.2025.1697161)
Supplement: Supplementary file 1 [file Supplementaryfile1.docx]

| Section |  | Questions |  |  | Where located |
| --- | --- | --- | --- | --- | --- |
|  |  |  | Control Group (Enhanced standard of care)* | Intervention Group | Primary paper |
| Brief name | 1 | Provide the name or a phrase that describes the intervention | Digital linkage of clinical data on pregnant women collected in the community and health care facilities | Digital linkage of clinical data on pregnant women collected in the community and health care facilities, and use of the linked data by work improvement teams to improve antenatal care uptake and services | Page 3 |
| Why | 2 | Describe any rationale, theory, or goal of the elements essential to the intervention. | Data are collected by community health promoters at the household level, but it has limited use for health care decision-making and quality improvement because they are not easily accessible to the healthcare providers at facilities offering clinical services to these communities. The trial involves maintaining digital linkage of the community and facility data, and for the intervention group, will train quality improvement teams that will access these linked data and use it for implementing quality improvement interventions. Implementation will take a phased format based on the plan-do-study-act cycle with periodic collaborative sessions to enable shared learning among the quality improvement teams. | | Pages 2 and 10 |
| What | 3 | Materials: Describe any physical or informational materials used in the intervention, including those provided to participants or used in intervention delivery or in training of intervention providers | Electronic Community Health Information System (eCHIS) application: This is a software application installed on the mobile phones assigned to each community health promoter (CHP) that enables electronic data capture at the community level. It has capability for household registration, pregnancy registration, service delivery, client referral to healthcare facilities and reminders for client follow up. Mobile phones with this application will be allocated to all CHPs and their supervisors (community health assistants) in the study area. | In addition to deployment of eCHIS, KenyaEMR and the linkage module, Work Improvement Teams (WITs) will be constituted and trained. Training material will be developed based on trial specific objectives and will be delivered through in-person training sessions. Data to inform antenatal care priority setting will be drawn from the National Guidelines on Quality Obstetrics and Perinatal Care (February 2022 edition).  Coaching and mentorship with be provided by facilitation team composed of project and sub-County/County health management team staff. | Pages 3 and 4 |
|  |  |  | KenyaEMR: This is an electronic medical records (EMR) system built on the Open MRS platform. The Maternal and Child Health (MCH) module which captures antenatal clinic, delivery and postnatal clinic data will be used by the clinical staff at all the trial sites. |  |  |
|  |  |  | Linkage module: This is a software application that will process client referrals from eCHIS to the facility EMR (KenyaEMR) and back to eCHIS using unique patient identifiers. |  |  |
|  |  | Provide information on where the materials can be accessed (e.g. online appendix, URL). | 1. eCHIS software and description: https://play.google.com/store/apps/details?id=org.medicmobile.webapp.mobile.moh_kenya_echis&pli=1 2. KenyaEMR software description: https://elearning.health.go.ke/enrol/index.php?id=44 3. Linkage module: https://linkage.goldindata.org/ | |  |
|  | 4 | Procedures: Describe each of the procedures, activities, and/or processes used in the intervention, including any enabling or support activities. | Mobile phones (with eCHIS installed) and log in credentials will be provided to CHPs and CHAs in all the trial sites. Training on the use of eCHIS will be provided in a standardised manner across all the sites. Tablets and log in credentials for KenyaEMR will be provided in all the trial sites and training on its use will be provided in a standardized manner across all the sites. Linkage module training will be conducted across all the trial sites and onsite technical support provided by the software developers. | Training of the WITs (the teams delivering the intervention) will take place in 3 phases following an improvement collaborative approach. The first phase will involve constitution of the WITs, drafting their terms of reference, training on the trial, training on 8 ANC contacts, priority ANC indicators and training on the community follow-up tool (a questionnaire on the experiences of pregnant women during ANC clinic visits and home CHP visits).  In the next phase, WITs will learn about accessing, summarizing and analysing data needed to monitor their indicators. They will review the data collected in the community follow-up tool and eCHIS to conduct a root cause analysis on the performance gaps and develop change plans. Subcounty health administration members will be trained as coaches for the WITs.  The last phase will involve 6 monthly learning events during which WITs, and county administration will convene to share experiences and challenges, and reward best practices. | Pages 3 and 4 |
| Who provided | 5 | For each category of intervention provider (e.g. psychologist, nursing assistant), describe their expertise, background and any specific training given. |  | The WITs will comprise: Community Health Promoter (community member serving in a community health unit after training on basic and technical modules**) Community Health Assistant (county government employee with training on community health modules*** who links the community unit and the local health facility) Healthcare facility in-charge (nurse trained in Nursing and Midwifery at diploma/degree level or clinical officer trained in Clinical Medicine at diploma or higher diploma level) ANC staff (nurse trained in Nursing and Midwifery at diploma/degree level or clinical officer trained in Clinical Medicine at diploma or higher diploma level) Adolescent representative (adolescent with no specific training) Community representative (adult with no specific training chosen by the community unit as a representative) | Page 4 |
| How | 6 | Describe the modes of delivery (e.g. face-to-face or by some other mechanism, such as internet or telephone) of the intervention and whether it was provided individually or in a group. |  | WITs will develop different interventions tailor-made for their context and challenges with a mode of delivery specific to the intervention e.g. holding community meetings to sensitize community members on ANC uptake. | Page 4 |
| Where | 7 | Describe the type(s) of location(s) where the intervention occurred, including any necessary infrastructure or relevant features. |  | WIT interventions will be deployed either in the community (through community health promoters and community representatives) or at the healthcare facilities. | Page 4 |
| When and How much | 8 | Describe the number of times the intervention was delivered and over what period of time including the number of sessions, their schedule, and their duration, intensity or dose. |  | WITs will be meeting monthly to review progress towards achieving their target indicators. The WITs will develop and deploy interventions unique to their setting which may be deployed continuously or at specific time-points based on the nature of the intervention. | Page 4 |
| Tailoring | 9 | If the intervention was planned to be personalised, titrated or adapted, then describe what, why, when, and how. |  | The WITs will maintain similar constitution across the intervention sites. | Page 4 |
| Modifications | 10 | If the intervention was modified during the course of the study, describe the changes (what, why, when, and how). |  | Will be described at the end of the study | N/A |
| How well | 11 | Planned: If intervention adherence or fidelity was assessed, describe how and by whom, and if any strategies were used to maintain or improve fidelity, describe them. |  | Will be described at the end of the study | N/A |
|  | 12 | Actual: If intervention adherence or fidelity was assessed, describe the extent to which the intervention was delivered as planned. |  | Will be described at the end of the study | N/A |
|  | | | | | |
| * A description is provided for the control sites since an enhanced standard of care will be implemented here. | | | | | |
| ** Basic modules: (i) health and development in the community, (ii) community governance and leadership, (iii) communication, advocacy and social mobilization, (iv) best practices for health promotion and disease prevention, (v) basic healthcare and life saving skills, and (vi) management and use of community health information and community disease surveillance. Technical modules: (i) integrated community case management, (ii) water, sanitation, and hygiene, (iii) maternal and newborn care, (iv) family planning, (v) HIV, TB and Malaria, (vi) community nutrition, and (vii) noncommunicable diseases. | | | | | |
| *** Modules: (i) health and development, (ii) governance and leadership, (iii) advocacy, communication and community engagement, (iv) health promotion, (v) disease prevention, (vi) basic health services, (vii) community health information and surveillance, and (viii) community health commodity management. | | | | | |
| CHP – Community Health Promoter, CHA – Community Health Assistant, WIT – Work Improvement Team, eCHIS – electronic Community Health Information System | | | | | |
